# Clinical and procedural outcomes of ostial left anterior descending artery intervention with or without crossover to left-main

**DOI:** 10.1186/s43044-026-00777-w

**Published:** 2026-09-13

**Authors:** Mohamed Sayed, Yasutsugu Shiono, Doaa A. Fouad, Ahmed Abdel-Galeel, Rikuya Tanaka, Shin Murakami, Satoshi Hata, Keisuke Yamamoto, Keiya Komatsu, Yuta Takano, Yoshinori Asae, Hiroki Emori, Akira Taruya, Masahiro Takahata, Teruaki Wada, Shingo Ota, Yuichi Ozaki, Manabu Kashiwagi, Akio Kuroi, Takashi Yamano, Hironori Kitabata, Atsushi Tanaka, Aly Tohamy

**Affiliations:** 1 Assiut University, Asyut, Egypt; 2 Wakayama Medical University, Wakayama, Japan

**Keywords:** Percutaneous coronary intervention, Ostial left anterior descending lesion stenting, left main crossover stenting, Intravascular imaging guidance, Major adverse cardiac events

## Abstract

**Background:**

It remains debated whether just ostial stenting (JOS) or crossover stenting (COS) is optimal for isolated ostial lesions of the left anterior descending (LAD) artery, and available comparative data on intravascular imaging (IVI)-guided percutaneous coronary intervention (PCI) remains limited. We aim to compare clinical outcomes between the strategies and identify determinants of strategy selection.

**Methods and results:**

This retrospective study included 172 patients with isolated ostial LAD lesions who underwent IVI-guided PCI with either JOS (*n* = 112) or COS (*n* = 60) between 2018 and 2025. The primary endpoint was two-year major adverse cardiac events (MACEs), defined as all-cause mortality, myocardial infarction, and any revascularization. MACE rates did not differ significantly in both strategies (23% vs.17%; adjusted hazard ratio: 0.74; 95% confidence interval [CI]: 0.37–1.49; *P* = 0.40). LAD diameter on angiography (odds ratio [OR] 2.04, 95% CI 1.03–4.05, *P* = 0.040) and LAD proximal reference area measured by IVI (OR 1.26, 95% CI 1.02–1.55, *P* = 0.033) were independently associated with selecting JOS over COS. Optimal minimum stent areas (MSA) were achieved in most cases in both groups (89% vs. 98%, *P* = 0.032).

**Conclusions:**

Under IVI-guided PCI, clinical outcomes showed no significant difference between JOS and COS, and LAD anatomy determined the strategy. These findings support individualized, anatomy-driven technique selection and IVI-guided procedural optimization for ostial LAD lesions.

**Supplementary Information:**

The online version contains supplementary material available at https://doi.org/10.1186/s43044-026-00777-w.

## Introduction

Ostial left anterior descending (LAD) artery lesions are particularly challenging for coronary artery disease intervention because of their impact on a large myocardial area and the technical demands of PCI [1]. Understanding the anatomical complexities and mastering stenting techniques specific to those lesions are essential for their management. Diagnostic challenges in ostial LAD stenting include difficulty in precisely delineating the ostial plane on angiography. Two-dimensional angiographic imaging provides limited information about the exact lesion location, making it difficult to deploy a stent with the proximal edge landing exactly at the ostium without strut protrusion into the left main coronary artery (LMCA) or incomplete coverage of the ostial stenosis. On the other hand, stent placements involving the LMCA bifurcation require complex procedures [2]. Either just ostial stenting (JOS) or crossover stenting (COS) is often used, but the best revascularization strategy is still debated, as highlighted in the European Bifurcation Club’s (EBC) expert consensus report [3]. 

Intravascular imaging (IVI) is key for guiding PCI in complex coronary lesions because it is superior to angiography alone in characterizing vessel anatomy, quantifying plaque burden, and assessing stent optimization [4]. Large randomized trials have consistently demonstrated that integrating IVI into routine PCI practice for such complex lesions improves clinical outcomes [5]. Ostial LAD lesions are expected to be among the subsets of coronary lesions that derive substantial benefit from IVI during PCI [2]. However, previous studies comparing JOS and COS strategies did not routinely incorporate their use. Consequently, this study aims to compare the clinical outcomes between the JOS and COS strategies in IVI-guided PCI for ostial LAD lesions.

## Materials and methods

### Study design and population

**Fig. 1:**
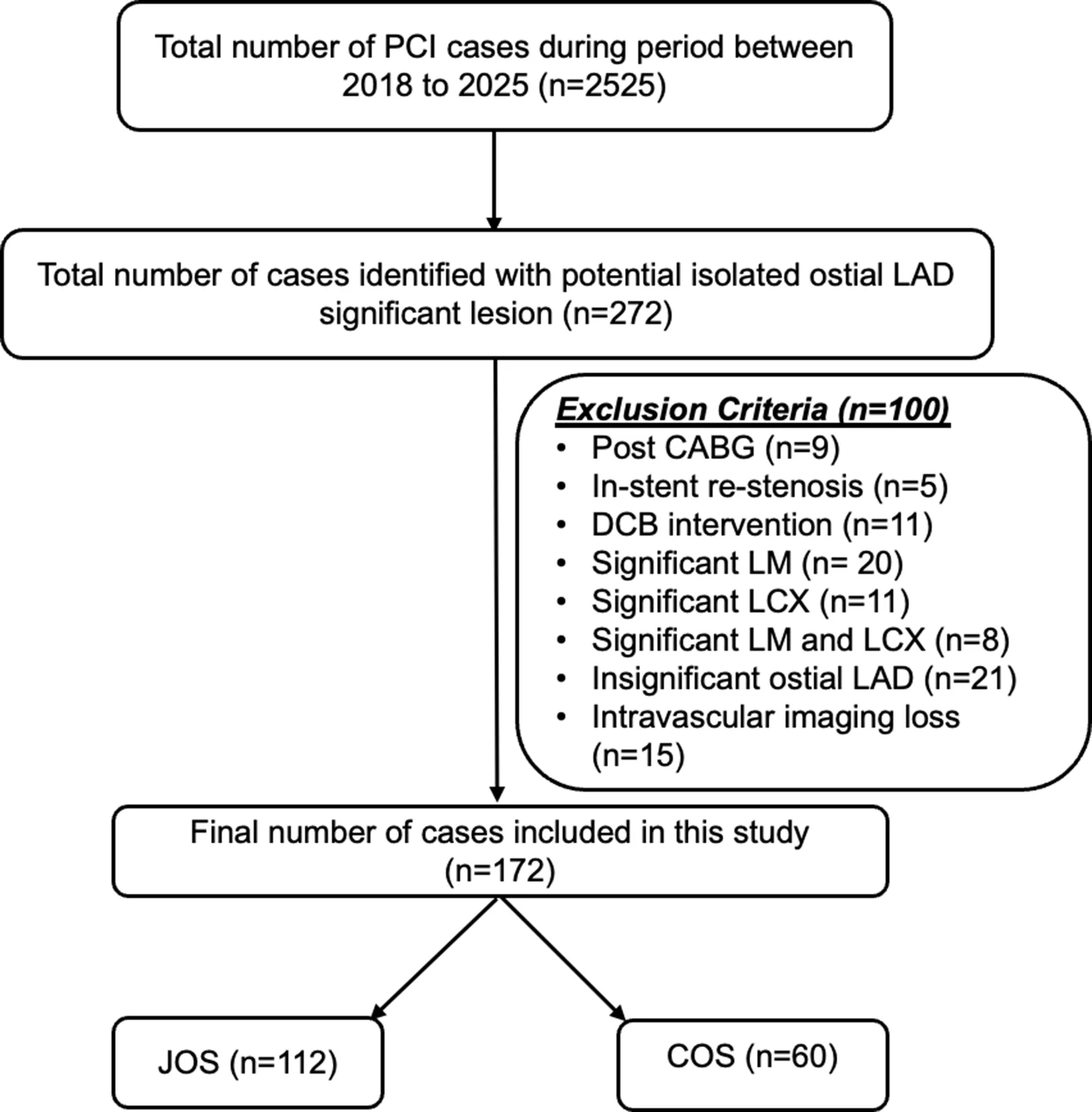
Patient flow chart. CABG, coronary artery bypass graft; COS, crossover stenting; DCB, drug-eluting balloon; LAD, left anterior descending artery; LCX, left circumflex artery; LM, left main artery; PCI, percutaneous coronary intervention; JOS, just ostial stenting

This retrospective single-center study investigated patients who underwent PCI for significant ostial LAD lesions (Medina 0,1,0) at our hospital between 2018 and 2025. We included intervention cases of ostial LAD lesions, defined as lesions within 3 mm of the LAD ostium with > 70% stenosis or a significant fractional flow reserve (≤ 0.80). The exclusion criteria were significant distal LMCA > 50%, significant ostial left circumflex (LCX) > 50%, post-coronary artery bypass graft (CABG) surgery, in-stent restenosis, or drug-eluting balloon (DCB) intervention only. Patients were divided into two groups by stenting strategy: those with stenting starting from the ostial LAD were classified as the just ostial stenting (JOS) group, and those with stenting from the LMCA crossover to the LAD were classified as the crossover stenting (COS) group, as illustrated in the study flow chart. (Fig. 1). Patient details were retrieved from the Hospital Information System database, including patient demographics, clinical and procedural details, devices used, and procedural outcomes. Written informed consent was waived due to the retrospective study design, which uses the data available in the electronic medical records.

### Coronary angiography and PCI

**Fig. 2:**
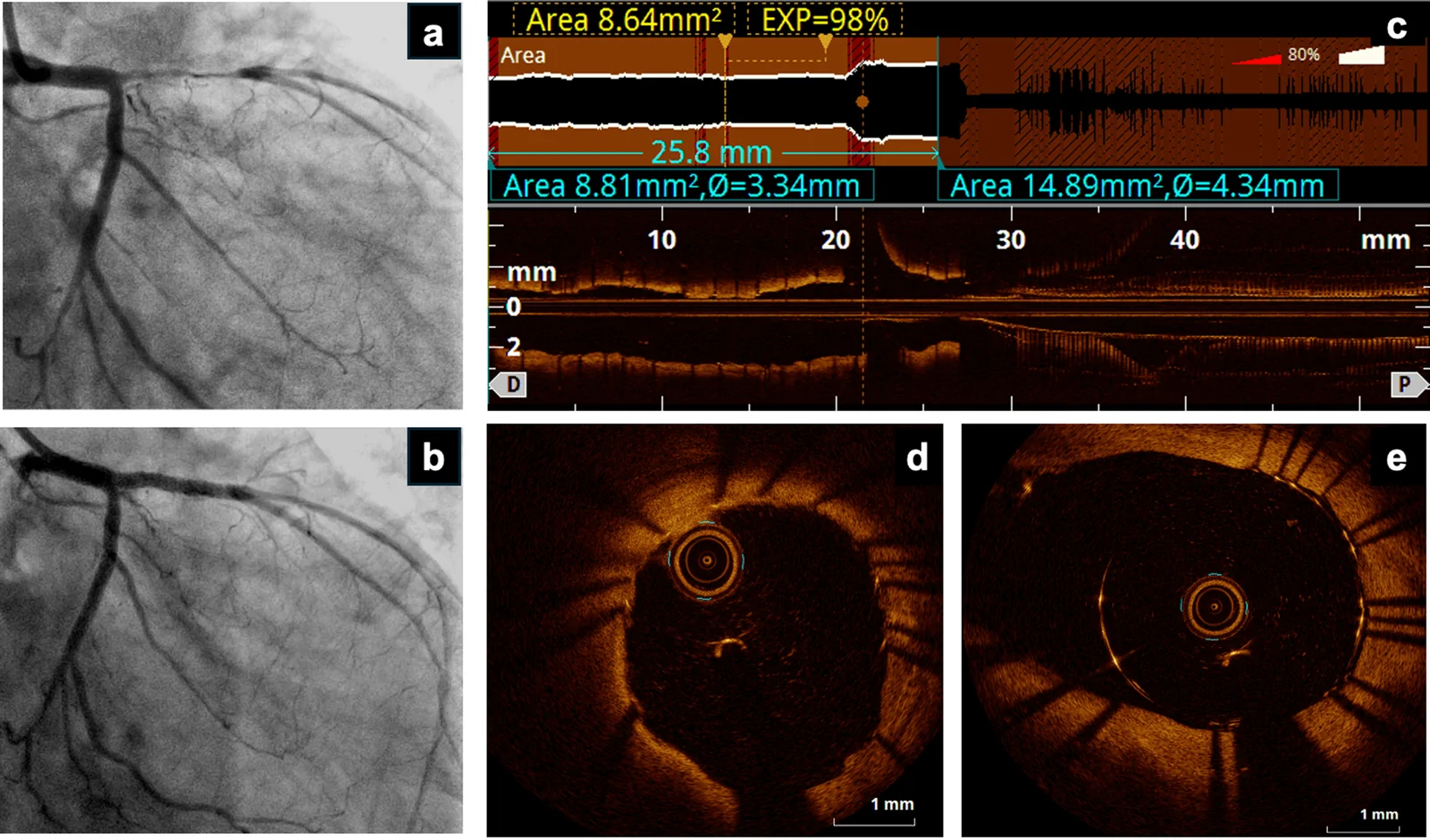
Representative case from the COS group. (**a, b**) angiographic view showing pre-PCI (**a**) and post-PCI (**b**), (**c, d, e**) OCT pullback showing(**c**) longitudinal view of the ostial LAD stent, (**d**) MSA at proximal LAD, (**e**) stent edge at LAD ostium. PCI percutaneous coronary intervention; OCT optical coherence tomography; MSA minimum stent area

**Fig. 3:**
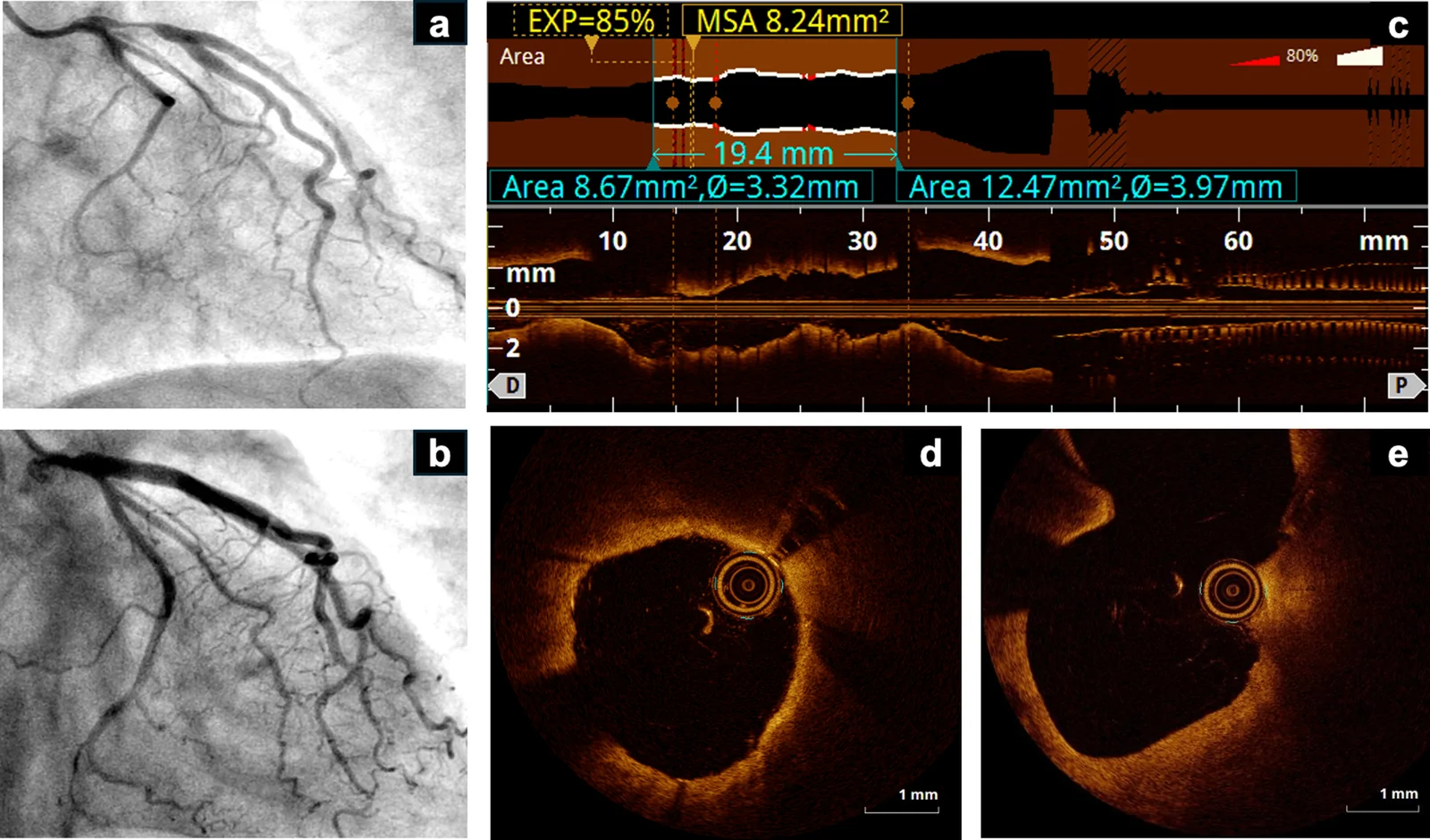
Representative case from the JOS group. (**a, b**) angiographic view showing pre-PCI (**a**) and post-PCI (**b**), (**c, d, e**) OCT pullback showing(**c**) longitudinal view of the ostial LAD stent, (**d**) MSA at proximal LAD, (**e**) stent edge at LAD ostium. PCI percutaneous coronary intervention; OCT optical coherence tomography; MSA minimum stent area

Each patient initially underwent a standard coronary angiogram via the radial or femoral artery. Before intervention, patients received aspirin (a 300 mg loading dose and/or a 100 mg daily maintenance dose) and either clopidogrel (a 300 mg loading dose and/or a 75 mg daily maintenance dose) or prasugrel (a 20 mg loading dose and/or a 3.75 mg daily maintenance dose). During the procedure, intravenous unfractionated heparin was administered at 70–100 IU/kg to maintain an activated clotting time > 250 s. In terms of PCI procedures, there were no specific criteria for strategy selection; approach site, stenting strategy for ostial LAD lesions, device choice, and stent optimization were totally at the operator’s discretion. Second- or third-generation drug-eluting stents were used in all cases. For the COS group, a stent was deployed from the LMCA into the LAD, as shown in Fig. 2, in accordance with EBC recommendations [3]. For the JOS group, the stent was carefully aligned with the LAD ostium without extending into the LMCA, as shown in Fig. 3. Both angiography and IVI were used to guide precise placement. Pre-and post-PCI coronary angiograms were analyzed using an off-line validated quantitative coronary angiography (QCA) bifurcation software (CASS Workstation version 7.3, Pie Medical) to measure LMCA, LAD, and LCX diameters (defined as the lumen diameter measured five mm distal or proximal from the polygon of confluence), bifurcation angle A (defined as the angle between LM and LCX) and B (defined as the angle between LAD and LCX), stenosis degree (%), and target lesion length.

### Intravascular imaging protocol

#### Optical coherence tomography (OCT) protocol

OCT was performed before and after PCI following intracoronary nitroglycerin administration (100–200 µg) using frequency-domain OCT systems (ILUMIEN OPTIS, Abbott Vascular, Santa Clara, CA, USA, or Optical Frequency Domain Imaging System, Terumo Corporation, Tokyo, Japan). A 2.7-Fr Dragonfly OPTIS or 2.6-Fr Fastview catheter was advanced over a guidewire, followed by automated pullback at 18–36 mm/s and 180 frames/s (Dragonfly OPTIS) or 20–40 mm/s and 160 frames/s (Fastview). Continuous contrast or low-molecular-weight dextran injection (3–4 mL/s) was used to visualize segments extending 10 mm proximal and distal to the lesion. OCT images were analyzed using an off-line proprietary software according to the latest expert consensus [6]. 

#### Intravascular ultrasound (IVUS) protocol

IVUS was performed before and after PCI after intracoronary nitroglycerin administration (100–200 µg). An IVUS catheter (AltaView, Terumo Corporation, Tokyo, Japan) was advanced at least 10 mm distal to the lesion and withdrawn at least 10 mm proximal to the lesion using motorized pullback at 0.5–9 mm/s. IVUS images were analyzed using off-line proprietary software in accordance with the latest expert consensus [7]. 

### Intravascular imaging assessment

Pre- and post-stent intravascular imaging were assessed according to the Expert Consensus Documents [6–11], which included proximal/distal reference area, minimum luminal area (MLA), average reference lumen area, stenosis degree, dominant lesion tissue characterization, minimum stent area (MSA), stent expansion index (SEI), major dissection, stent malapposition, and stent struts protrusion. MSA was measured once in the LAD for the JOS group, while it was measured at three points (LAD, the confluence of the LAD and the LCX, and the LMCA), and the smallest of the three was selected for the COS group according to the EBC recommendation [8]. SEI was measured once for the JOS group and twice for the COS group by dividing the vessel into two segments at the carina, with the smaller SEI selected [9]. 

### Clinical endpoints

The primary endpoint was two-year major adverse cardiovascular events (MACE), defined as a composite of all-cause mortality, any myocardial infarction (MI), and any revascularization. Secondary endpoints included each MACE component, cardiovascular and non-cardiovascular death, target-vessel and non-target-vessel MI, target-lesion revascularization (TLR), target-vessel revascularization (TVR), stent thrombosis, and any stroke. All event definitions followed the Academic Research Consortium (ARC-2) criteria [12]. In-hospital outcomes and complications were documented at discharge or in-hospital mortality. Long-term follow-up data, collected during outpatient clinic visits or by telephone as part of clinical practice, were obtained retrospectively from electronic health records.

### Statistical analysis

Categorical variables were presented as counts and percentages and compared using the chi-square test or Fisher’s exact test, as appropriate. Continuous variables were presented as medians with interquartile ranges (IQRs) and compared using the Mann-Whitney U test. For survival analysis, sample size was calculated based on previously reported two-year MACE rates of 28.2% in the COS group and 8.2% in the JOS group [13]. Assuming a 1:1 allocation ratio, a minimum of 110 patients (55 per group) was required to achieve 80% power at a two-sided alpha level of 0.05 using G*Power version 3.1.9.7. Kaplan-Meier analysis was used to assess clinical endpoints, with group comparisons performed using the Log-Rank test. Age- and sex-adjusted hazard ratios (aHRs) were estimated using Cox proportional hazards models for clinical outcomes. As a sensitivity analysis, propensity score matching was also performed to minimize baseline differences using a logistic regression model with baseline clinical variables, including sex, prior PCI, left ventricular ejection fraction (LVEF), and clinical presentation. Nearest-neighbor 1:1 matching without replacement was applied, and covariate balance was assessed by comparing baseline characteristics between matched groups. Factors associated with technique selection (COS vs. JOS) were evaluated using univariable logistic regression. Variables with *P* < 0.10 in univariable analysis, together with clinically relevant variables, were included in multivariable logistic regression models. Results are reported as odds ratios (ORs) with 95% confidence intervals (CIs). All tests were two-sided, and *P* < 0.05 was considered statistically significant. Statistical analyses were performed using IBM SPSS Statistics version 27 (IBM Corp., Armonk, NY).

## Results

### Study population

Among 2,525 patients who underwent PCI during the study period, 272 were identified as potential ostial LAD PCI cases. Two interventional cardiologists reviewed them and applied the inclusion and exclusion criteria (Fig. 1). Patients who had the following conditions were excluded: distal LMCA lesion > 50% (*n* = 20), ostial LCX lesion > 50% (*n* = 11), both distal LMCA and ostial LCX lesions (*n* = 8), insignificant ostial LAD disease (*n* = 21), prior CABG (*n* = 9), in-stent restenosis (*n* = 5), DCB-only intervention (*n* = 11), and missing IVI data (*n* = 15). The final analysis included 172 patients, exceeding the calculated sample size of 110 cases.

### Patient demographics

A total of 172 eligible patients were categorized into the JOS group (*n* = 112) and COS group (*n* = 60). As shown in Table 1, baseline demographic and clinical characteristics, including age, sex, BMI, and cardiovascular comorbidities (diabetes, hypertension, dyslipidaemia), were generally comparable between the groups. However, STEMI presentation was more frequent in the JOS group (46% vs. 28%, *P* = 0.021), and the JOS group had a lower median LVEF than the COS group (49.2% [IQR 41.5–57] vs. 56.7% [IQR 44.3–58.7], *P* = 0.027). Baseline laboratory findings were similar between groups.

### Angiographic and PCI procedural findings

Angiography and PCI data are summarized in Table 2. Radial access was common in both groups, but larger sheaths were more often used in the COS group than in the JOS group (sheath size ≥7Fr: 88% vs. 61%; *P* < 0.001). QCA data showed that the COS group had significantly smaller LMCA (3.7 [3.3–4.1] mm vs. 3.9 [3.5–4.5] mm, *P* = 0.030) and LAD diameters (2.1 [1.6–2.5] mm vs. 2.4 [2.2–2.8] mm, *P* < 0.001) than the JOS group. The COS group showed a larger stent diameter (3.5 [3.5–3.5] mm vs. 3.0 [3.0-3.5] mm, *P* < 0.001) and a higher rate of two-stent use (22% vs. 9%, *P* = 0.019) than the JOS group. Post-stent balloon dilation was more frequent in the COS group than in the JOS group (97% vs. 69%, *P* < 0.001), with a larger balloon size (4.5 [4.0–5.0] mm vs. 3.5 [3.2-4.0] mm, *P* < 0.001) but a lower balloon inflation pressure (14 [12–19] atm vs. 20 [14–22] atm, *P* < 0.001). The COS group showed significantly greater use of complex bifurcation techniques, including LCX protection wire (98% vs. 83%, *P* = 0.003), kissing balloon inflation [KBI] (57% vs. 1%, *P* < 0.001), and proximal optimization technique [POT] (92% vs. 5%, *P* < 0.001). Procedural duration was longer (125 [100–150] min vs. 96 [70–132] min, *P* < 0.001), and fluoroscopy dose was higher (2238 [1443–3038] mGy vs. 1694 [1187–2482] mGy, *P* = 0.012) in the COS group compared to the JOS group; however, contrast volume did not significantly differ (155 [128–190] mL vs. 140 [110–180] mL, *P* = 0.06) between the two groups.

### Intravascular imaging findings

Intravascular imaging findings are summarized in Table 3. OCT was used more frequently than IVUS in both groups, with no significant differences between-group differences (COS: 70% vs. 30%; JOS: 82% vs. 18%; *P* = 0.07). Lesion tissue characteristics differ significantly (*P* = 0.032), with more calcified lesions in the COS group (38% vs. 21%) and more thrombotic lesions in the JOS group (24% vs.10%). Pre-PCI imaging measurements were generally comparable, except for a smaller OCT-derived proximal reference area in the COS group (5.4 [4.4–6.6] mm² vs. 6.8 [5.7–8.5] mm², *P* < 0.001). In post-PCI IVI findings, the COS group showed lower minimal SEI (76.7% [71.2–80.9] vs. 84.0% [80.9–92.2], *P* < 0.001) and LAD SEI (81.9% [78.9–86.6] vs. 84.0% [80.9–92.2], *P* = 0.033), despite comparable MSA between the groups (OCT-measured MSA: 6.0 [5.4–6.7] mm^2^ vs. 5.9 [5.1–7.3] mm^2^, *P* = 0.74, and IVUS-measured MSA: 6.3 [5.6–7.0] mm^2^ vs. 6.6 [5.6–7.8] mm^2^, *P* = 0.57). Stent malapposition was more frequent in the COS group (12% vs. 3%, *P* = 0.016), whereas stent protrusion into the LCX (0% vs. 11%, *P* = 0.009) and LMCA (0% vs. 7%, *P* = 0.034) occurred more often in the JOS group. Achievement of SEI > 80% was less frequent in the COS group (33% vs. 83%, *P* < 0.001), while MSA targets were achieved more often in the COS group (98% vs. 89%, *P =* 0.032).

### Factors related to the selection of JOS over COS

Univariable analyses identified several variables associated with selection of the JOS, including STEMI, LVEF, LMCA diameter, LAD diameter, LAD stenosis degree, and proximal reference area. In multivariable logistic regression, LAD diameter measured by QCA (OR 2.04, 95% CI 1.03–4.05, *P* = 0.040) and proximal reference area measured by IVI (OR 1.26, 95% CI 1.02–1.55, *P* = 0.033) were independently associated with selection of the JOS technique (Table 4**)**.

### Peri-procedural complications

The peri-procedural complications are shown in Supplementary Table 1, with no significant differences across groups.

### Two-year clinical outcomes

**Fig. 4:**
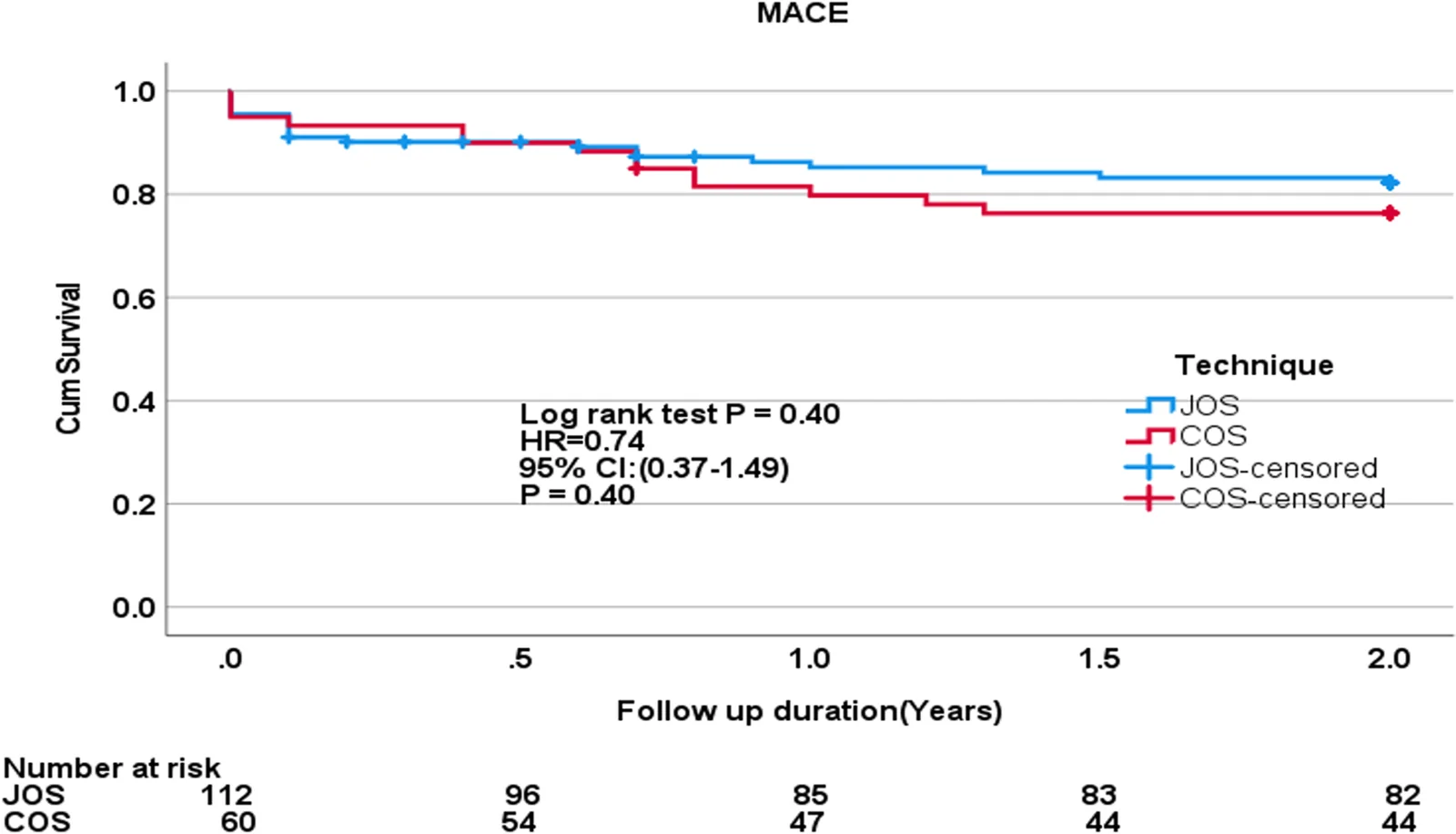
Kaplan Meier Curve for MACE. CI confidence interval; COS crossover stenting; HR hazards ratio; MACE major adverse cardiac event; JOS just ostial stenting

**Fig. 5:**
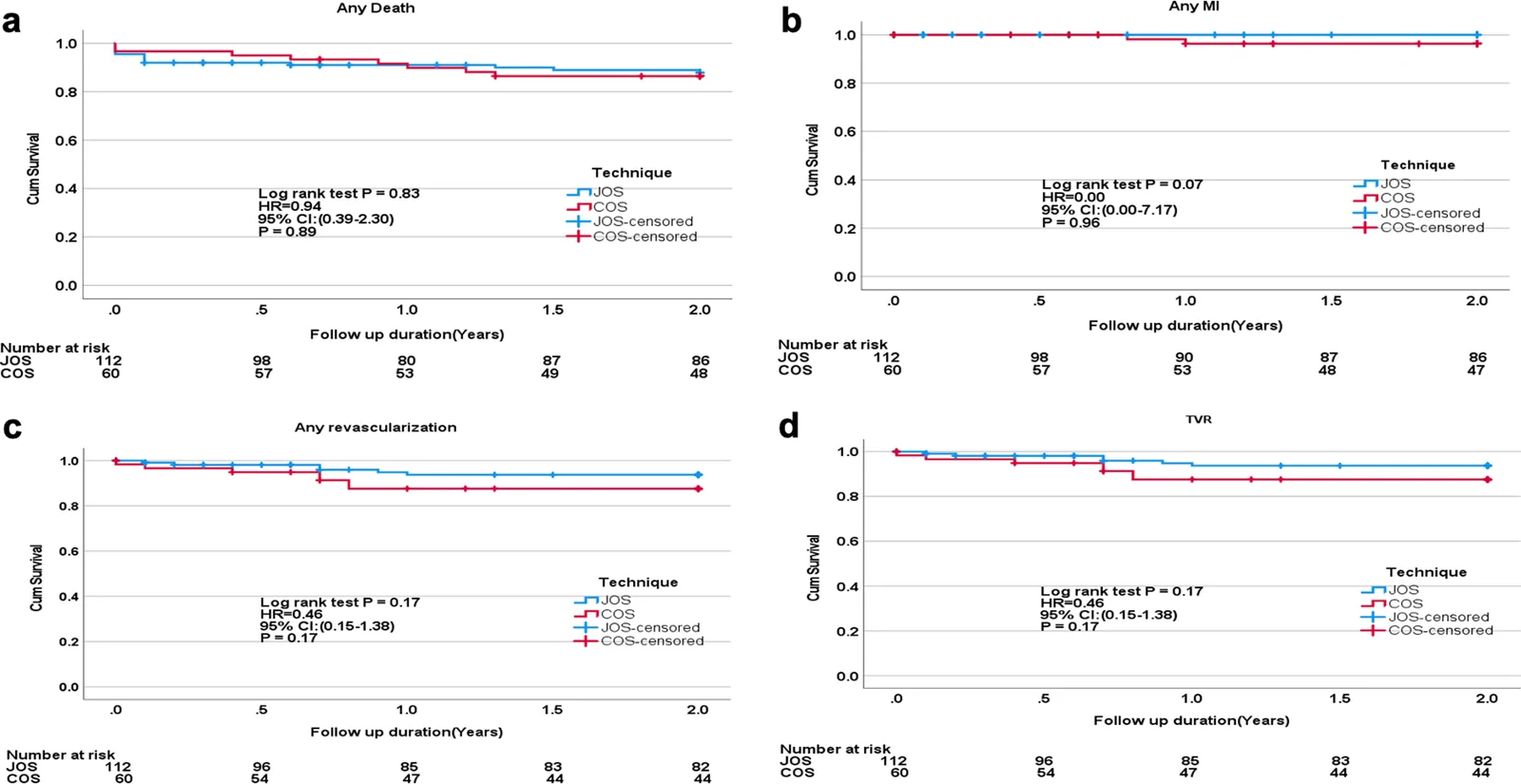
Kaplan Meier Curve for components of MACE and TVR. CI confidence interval; COS crossover stenting; HR hazards ratio; MACE major adverse cardiac event; MI myocardial infarction; TVR target vessel revascularization; JOS just ostial stenting

Two-year follow-up rates were 93.3% in the COS group and 88.4% in the JOS group. The incidence of MACE did not differ significantly between the COS and JOS groups (23% vs. 17%; aHR: 0.74; 95% CI: 0.37–1.49; *P* = 0.40) (Fig. 4). Similarly, no significant differences were observed in the incidence of all-cause mortality (13% vs. 12%, aHR: 0.94, 95% CI: 0.39–2.30, *P* = 0.89), any MI (3% vs. 0%, aHR: 0.00, 95% CI: 0.00–7.17, *P* = 0.96), any revascularization (12% vs. 5%, aHR: 0.46, 95% CI: 0.15–1.38, *P* = 0.17), and TVR (12% vs. 5%, aHR: 0.46, 95% CI: 0.15–1.38, *P* = 0.17) (Fig. 5 and Supplementary Table 2).

Also, no statistical significant difference in the clinical outcomes were observed in the propensity score matching analysis, which generated 56 matched pairs (112 patients) (Supplementary Table 3): the incidence of MACE (23% vs.13%, *P* = 0.21), all-cause mortality (14% vs. 9%, *P* = 0.48), any MI (2% vs. 0%, *P* = 0.34), any revascularization (11% vs. 4%, *P* = 0.18), and TVR (11% vs. 4%, *P* = 0.18) (Supplementary Figs. 1 and 2).

## Discussion

This study assessed 2-year clinical outcomes of JOS and COS strategies in patients with isolated ostial LAD lesions (Medina 0,1,0) undergoing IVI-guided PCI, as well as factors associated with the selection of these techniques. Our main findings are summarized as follows: (1) MACE rates did not differ significantly between the two strategies, (2) post-PCI IVI findings revealed differences in relative stent expansion and in the incidence of stent malapposition and stent protrusion, but MSA was comparable, and (3) LAD vessel size measured by angiography and IVI was the determinant of technique selection.

### Insignificant difference in clinical outcomes between strategies under IVI-guided PCI

The optimal PCI strategy for isolated ostial LAD lesions remains controversial, as both COS and JOS have distinct advantages and limitations. COS minimizes the risk of geographic miss but requires more complex management of the LMCA-LAD bifurcation. In contrast, JOS is technically simpler but carries a risk of geographic miss, which can result in incomplete plaque coverage or stent protrusion into the bifurcation. Previous studies reported conflicting results regarding the clinical outcomes of the two strategies. For example, Soylu et al. reported two-year MACE rates of 28.2% in the COS and 8.2% in the JOS [13], whereas Tanik et al. reported rates of 9.5% in the COS versus 18.1% in the JOS during a mean follow-up of 2.5 years [14]. To settle these conflicts, Han Shi et al. conducted a systematic review and meta-analysis of seven studies comprising 1,118 patients and demonstrated that COS was superior to JOS in terms of TLR (OR 0.51, 95% CI 0.30–0.86, *P* = 0.01), but neither in cardiovascular mortality (OR 0.86, 95% CI 0.17–4.32, *P* = 0.85) nor in MI (OR 0.83, 95% CI 0.31–2.24, *P* = 0.71). In addition, they revealed that IVUS use negated the difference in TLR rates between the strategies (OR 0.85, 95% CI 0.41–1.76, *P* = 0.66), suggesting that either strategy can yield comparable MACE rates when PCI is guided by IVI [15]. This aligns with our analysis of IVI-guided PCI, which showed no statistically significant difference in MACE, including all-cause mortality, any MI, and any revascularization, between the two strategies at the two-year follow-up. Our results are also consistent with Yamamoto et al., who, in an IVUS-guided PCI study of ostial LAD lesions comparing 30 patients undergoing COS with 46 patients undergoing JOS in the acute MI setting, reported no significant difference in MACE during the mean follow-up period of 578 days (16.7% vs. 21.7%, *P* = 0.587) [16]. While their study included only patients with acute MI and used IVUS exclusively, our study extended these findings by including both CCS and ACS patients and using both IVUS and OCT.

### Post-PCI IVI findings between COS and JOS in ostial LAD intervention

Stent optimization, as assessed by IVI, is essential for achieving a favorable clinical outcome. Several parameters, such as MSA, relative stent expansion, edge dissection, and malapposition, have been suggested to predict outcomes [10]. This study revealed distinct mechanistic differences between COS and JOS in the post-PCI IVI findings. Stent malapposition was more frequent in the COS than in the JOS group, whereas stent strut protrusion toward the LMCA or LCX was observed only in the JOS group. The clinical impact of these suboptimal imaging findings—malapposition in COS and protrusion in JOS—remains a subject of debate [17–19], Notably, our results showed that despite these differences, the incidence of MACE, MI, and stent thrombosis remained low and did not significantly differ between the groups. This suggests that the potential risks posed by these mechanical issues were likely mitigated by achieving sufficient MSA, which was similar across both strategies (COS: 98% vs. JOS: 89% meeting target criteria). These observations align with a secondary analysis of the RENOVATE-COMPLEX-PCI trial, which indicated that while various imaging parameters may correlate with target lesion failure, their prognostic impact is significantly attenuated when an optimal MSA is ensured [20]. Therefore, our findings reinforce the concept that ensuring an adequate stent scaffold area is the primary procedural determinant of long-term success, potentially overriding the minor mechanical drawbacks inherent to each specific technique. Future studies with larger cohorts are needed to further explore whether these specific abnormalities, such as strut protrusion in JOS, might influence very-late-term outcomes or specific events like late stent thrombosis.

### Determinants of COS or JOS strategies

Beyond clinical outcomes, our analysis provides important insights into the determinants of technique selection between JOS and COS for ostial LAD intervention. Our findings demonstrate that the choice was predominantly driven by anatomical factors rather than by demographic or clinical factors. Specifically, LAD diameter, assessed by QCA, and proximal reference area, measured by IVI, emerged as independent factors associated with JOS choice, indicating that vessel anatomy remains critical in procedural selection. These findings align with current recommendations that the choice of stenting technique should be based on anatomical geometry [3]. These results support a tailored, anatomy-based approach, in which operators preferentially adopt JOS when angiography shows a larger vessel caliber, and IVI shows a larger proximal reference area, while COS may be selected when LAD anatomy is unsuitable for JOS, and a safe landing zone in LMCA is available. This reinforces the concept that the optimal ostial LAD PCI strategy should be individualized based on lesion anatomical characteristics rather than relying on a uniform approach [21], particularly given the lack of significant differences in clinical outcomes between techniques.

### Advantages and disadvantages of JOS and COS strategies *(procedural considerations in JOS and COS strategies)*

Our findings suggest that JOS and COS should be viewed as complementary strategies with distinct procedural advantages rather than as competing techniques with differential clinical efficacy. As shown in Tables 2 and 3, JOS offers greater procedural simplicity, with lower fluoroscopy time, radiation exposure, and contrast volume. In contrast, COS requires a more complex procedural approach, including side-branch protection, wire recrossing, and additional KBT and POT, but achieved optimal MSA more frequently in our cohort. These differences in procedural characteristics are clinically relevant, as the simpler approach of JOS may reduce procedural burden and radiation exposure, whereas COS may provide greater control over stent coverage and expansion at the LAD ostium. However, these procedural differences did not translate into a significant difference in MACE between the two techniques. This finding is consistent with previous comparative studies and a recent meta-analysis, which have not demonstrated a consistent difference in MACE between ostial and crossover stenting strategies [22, 23]. Accordingly, the choice between JOS and COS should be individualized according to lesion and vessel characteristics, particularly LAD diameter, which may influence the feasibility and potential advantages of each approach. Within this anatomy-driven framework, IVI can facilitate accurate assessment of vessel dimensions and lesion morphology while guiding stent sizing, expansion, and final optimization. Thus, the practical advantages of procedural simplicity with JOS should be balanced against the potential for more extensive stent coverage and expansion with COS according to the individual anatomical characteristics of the LAD lesion.

### Limitation

Our study has several limitations. Firstly, this is a retrospective observational study conducted at a single center with a relatively small sample size. However, no randomized controlled trial has compared COS and JOS for isolated ostial LAD lesions, and our cohort was the largest among studies that exclusively assessed IVI-guided PCI [16] Secondly, because we including consecutive patients undergoing IVI-guided PCIs for isolated ostial LAD lesions during study period with minimal exclusion criteria, the cohort had diverse indications, including CCS and ACS, and those with OCT- and IVUS-guided PCIs. This inclusion approach made the study population as reflective of real clinical practice as possible, but it led to imbalances in baseline demographics and lesion characteristics, including the rate of prior PCI, LVEF, LMCA and LAD diameters, and the degree of LAD stenosis. The limited sample size precluded subgroup analyses and full adjustment for these imbalances in the Cox proportional hazards models and propensity score matching. Therefore, our findings need to be confirmed in larger studies or in a randomized controlled study.

## Conclusion

For isolated ostial LAD lesions, LAD anatomy determined whether JOS or COS techniques were used during PCI. Clinical outcomes show no significant difference between the two techniques when PCI was performed under IVI guidance, supporting individualized, anatomy-driven strategies using IVI for these lesions.

**Table 1:** Baseline demographic and clinical characteristics

	JOS (112)	COS (60)	*P* value
Age (years)	73 (66–80)	77 (64–81)	0.52
Male sex, N (%)	78 (70)	50 (83)	0.05
BMI	22.6 (20.7–25.4)	23.8 (21.7–25.7)	0.11
Hypertension, N (%)	78 (70)	46 (77)	0.33
Diabetes mellitus, N (%)	51 (46)	31 (52)	0.44
Dyslipidemia, N (%)	68 (61)	40 (67)	0.44
Smoking, N (%)			0.2
Never	58 (52)	30 (50)	
Current	25 (22)	8 (13)	
Former	29 (26)	22 (37)	
Family history, N (%)	25 (22)	19 (32)	0.18
Peripheral arterial disease, N (%)	3 (3)	1 (2)	0.68
Hemodialysis, N (%)	8 (7)	3 (5)	0.58
Prior MI, N (%)	10 (9)	11 (18)	0.73
Prior PCI, N (%)	19 (17)	18 (30)	0.047
Prior heart failure, N (%)	4 (4)	2 (3)	0.94
Clinical presentation, N (%)			0.11
CCS	40 (36)	29 (48)	
UA	8 (7)	4 (7)	
NSTEMI	12 (11)	10 (17)	
STEMI	52 (46)	17 (28)	
Cardiogenic shock, N (%)	9 (8)	4 (7)	0.77
LV ejection fraction (%)	49.2 (41.5–57.0)	56.7 (44.3–58.7)	0.027
Lab. Investigations			
Hemoglobin (g/dL)	13.4 (12.0–15.0)	13.6 (12.1–14.6)	0.87
Platelets (10^4/µL)	20.0 (16.1–24.6)	20.0 (16.3–25.6)	0.92
Creatinine (mg/dL)	0.90 (0.78–1.20)	0.96 (0.80–1.20)	0.33
HbA1c (%)	6.0 (5.6–6.9)	6.2 (5.7-7.0)	0.32
LDL-c (mg/dL)	98 (69–125)	91 (67–122)	0.23
Discharge medications, N (%)			
Aspirin	110 (98)	58 (97)	0.3
P2Y12 inhibitors	111 (99)	60 (100)	0.46
ACEIs/ARBs	86 (77)	44 (73)	0.62
BBs	75 (67)	31 (52)	0.41
Lipid lowering drugs	111 (99)	59 (98)	0.65
Nitrates	13 (12)	9 (15)	0.53
SGLT2i	12 (11)	11 (18)	0.16
CCBs	28 (25)	12 (20)	0.46
OAC	8 (7)	4 (7)	0.91

Values are N (%) or Median (Interquartile range). *ACEIs* angiotensin converting enzyme Inhibitors; *ARBs* angiotensin II receptor blockers; *BBs* beta blockers; *BMI b*ody mass index; *CCBs* calcium channel blockers; *CCS* chronic coronary syndrome; *HbA1c* glycated hemoglobin; *LDL-C* low-density lipoprotein cholesterol; *MI* myocardial infarction; *NSTEMI* Non-ST elevation myocardial infarction; O*AC* oral anticoagulation; *PCI p*ercutaneous coronary intervention; *SGLT2i* sodium glucose co-transport 2 inhibitors; *STEMI* ST elevation myocardial infarction; *UA* unstable angina

**Table 2:** Angiographic and PCI procedural data

	JOS (112)	COS (60)	*P* value
Access site, N (%)			0.73
Femoral	23 (21)	11 (18)	
Radial	89 (79)	49 (82)	
Sheath size (Fr), N (%)			< 0.001
6	44 (39)	7 (12)	
7	68 (61)	47 (78)	
8	0 (0)	6 (10)	
Pre stenting QCA data			
Bifurcation angle B (°)	82 (57–109)	83 (67–96)	0.72
Bifurcation angle A (°)	114 (93–134)	129 (108–147)	0.11
LMCA diameter (mm)	3.9 (3.5–4.5)	3.7 (3.3–4.1)	0.03
LCX diameter (mm)	2.7 (2.3–3.2)	2.5 (2.2–2.8)	0.06
LAD diameter (mm)	2.4 (2.2–2.8)	2.1 (1.6–2.5)	< 0.001
LAD lesion length (mm)	22.4 (16.0-31.2)	21.9 (16.4–30.6)	0.88
LAD stenosis (%)	77 (72–84)	77 (70–82)	0.043
Post stenting QCA data			
Residual stenosis (%)	9 (0–13)	9 (0–13)	0.99
Pre-dilation balloon size (mm)	2.8 (2.5-3.0)	2.6 (2.5-3.0)	0.57
Pre-dilation balloon inflation pressure (atm)	14 (12–18)	16 (13–20)	0.038
Thrombus aspiration, N (%)	10 (9)	4 (7)	0.61
Stent diameter (mm)	3.0 (3.0-3.5)	3.5 (3.5–3.5)	< 0.001
Stent length (mm)	24 (18–38)	28 (23–33)	0.25
Inflation degree (atm)	12 (11–16)	12 (11–16)	0.84
Post-stent dilation Use, N (%)	77(69)	58(97)	< 0.001
Post-stent dilation balloon diameter (mm)	3.5(3.2-4)	4.5(4–5)	< 0.001
Post-stent dilation balloon inflation (atm)	20(14–22)	14(12-18.5)	< 0.001
Number of stents in LAD, N (%)			0.019
1	102 (91)	47 (78)	
2	10 (9)	13 (22)	
LCX protection wire, N (%)	93 (83)	59 (98)	0.003
KBI, N (%)	1 (1)	34 (57)	< 0.001
POT, N (%)	5 (5)	55 (92)	< 0.001
Procedural duration (min.)	96 (70–132)	125 (100–150)	< 0.001
Fluoroscopy time (min.)	35.3 (24.2–51.0)	46.4 (33.7–61.8)	0.002
Fluoroscopy dose (mGy)	1694 (1187–2482)	2238 (1443–3038)	0.012
Contrast volume (ml)	140 (110–180)	155 (128–190)	0.06

Values are N (%) or Median (Interquartile range) *KBI* kissing balloon inflation; *LAD* left anterior descending artery; *LCX* left circumflex artery; *LMCA* left main coronary artery; *POT* proximal optimization technique; *QCA q*uantitative coronary angiography

**Table 3:** Pre- and post-PCI intravascular imaging findings

	JOS (112)	COS (60)	*P* value
Intravascular imaging used, N (%)			0.07
IVUS	20 (18)	18 (30)	
OCT	92 (82)	42 (70)	
Lesion tissue Characterization, N (%)			0.032
Lipid	23 (21)	11 (18)	
Fibrous	39 (34)	20 (33)	
Calcium	23 (21)	23 (38)	
Thrombus	27 (24)	6 (10)	
Pre-PCI OCT Measurements			
MLA (mm^2^)	1.1 (0.8–1.5)	1.2 (0.9–1.4)	0.81
Stenosis degree (%)	79.7 (74.0-84.3)	77.5 (73.0-82.1)	0.18
Distal reference area (mm^2^)	4.3 (3.3–5.7)	4.9 (3.9–6.1)	0.07
Proximal reference area (mm^2^)	6.8 (5.7–8.5)	5.4 (4.4–6.6)	< 0.001
Distance from MLA location to the distal LMCA	8.5 (7.5–9.4)	7.0 (6.5–8.5)	0.009
Pre-PCI IVUS Measurements			
MLA (mm^2^)	1.9 (1.6–2.5)	1.8 (1.6-2.0)	0.72
Stenosis degree (%)	71.1 (67.0-77.5)	71.2 (69.0-78.8)	0.57
Distal reference area (mm^2^)	5.4 (4.6–6.2)	5.2 (4.1-6.0)	0.39
Proximal reference area (mm^2^)	7.7 (6.3–8.8)	7.5 (6.5–8.2)	0.85
Distance from MLA location to the distal LMCA	8.5 (8.0-10.3)	6.5 (5.5–8.1)	< 0.001
Post-PCI intravascular imaging measurements			
MSA(OCT) (mm^2^)	5.9 (5.1–7.3)	6.0 (5.4–6.7)	0.74
MSA(IVUS) (mm^2^)	6.6 (5.6–7.8)	6.3 (5.6-7.0)	0.57
SEI LAD (%)	84.0 (80.9–92.2)	81.9 (78.9–86.6)	0.033
Minimal SEI (%)	84.0 (80.9–92.2)	76.7 (71.2–80.9)	< 0.001
Stent mal-apposition, N (%)	3 (3)	7 (12)	0.016
Major dissection, N (%)	6 (5)	2 (3)	0.55
Struts protrusion to LCX, N (%)	12 (11)	0 (0)	0.009
Struts protrusion to LMCA, N (%)	8 (7)	0 (0)	0.034
SEI > 80% of average reference area	93 (83)	20 (33)	< 0.001
MSA > 5.5 mm^2^ (IVUS), > 4.5 mm^2^ (OCT)	100 (89)	59 (98)	0.032

Values are N (%), or Median (Interquartile range). *IVUS* intravascular ultrasound; *LCX* left circumflex artery; *LMCA* left main coronary artery; *MLA* minimum luminal area; *MSA m*inimum stent area; *OCT* optical coherence tomography; *PCI* percutaneous coronary intervention; *SEI* stent expansion index

**Table 4:** Univariable and multivariable logistic regression analyses of independent factors for JOS versus COS techniques in patients with ostial LAD

	Univariable regression			Multivariable regression		
	OR	95% CI	*P* value	OR	95% CI	*P* value
Age	0.99	0.97–1.03	0.84			
Male sex	0.46	0.21–1.01	0.053	0.48	0.20–1.14	0.11
Prior PCI	0.48	0.23-1.00	0.050	0.62	0.26–1.44	0.26
STEMI	2.19	1.12–4.29	0.022	1.25	0.53–2.93	0.61
LV ejection fraction	0.97	0.94-1.00	0.048	0.98	0.95–1.02	0.32
Bifurcation angle B	1.00	0.98–1.01	0.93			
Bifurcation angle A	0.99	0.98-1.00	0.15			
LMCA diameter	1.69	1.04–2.76	0.034	1.17	0.76–1.80	0.48
LCX diameter	1.74	0.99–3.06	0.054	1.03	0.52–2.04	0.93
LAD diameter	2.45	1.37–4.35	0.002	2.04	1.03–4.0	0.040
LM-LAD diameter	0.98	0.69–1.39	0.93			
LAD stenosis	1.03	1.00-1.06	0.036	1.02	0.99–1.05	0.26
Lesion tissue characterization	1.08	0.79–1.48	0.61			
Distal reference area	0.87	0.73–1.03	0.11			
Proximal reference area	1.36	1.13–1.65	0.002	1.26	1.02–1.55	0.033

CI confidence interval; LAD left anterior descending artery; LCX left circumflex artery; LMCA left main coronary artery; OR odds ratio; PCI percutaneous coronary intervention; STEMI ST elevation myocardial infarction

## Supplementary Information

Below is the link to the electronic supplementary material.

Supplementary Material 1

## Data Availability

No datasets were generated or analysed during the current study.
