# Supplementary material for: Clinical and procedural outcomes of ostial left anterior descending artery intervention with or without crossover to left-main
Source: Egypt Heart J. 2026 Sep 13;78:65. doi: 10.1186/s43044-026-00777-w (PMC13572354; doi:10.1186/s43044-026-00777-w)
Supplement: Supplementary file 1 [file 43044_2026_777_MOESM1_ESM.docx]

**Supplementary Appendix**

**Title:** Clinical and procedural outcomes of ostial left anterior descending artery intervention with or without crossover to left-main

**Journal:** The Egyptian Heart Journal

**Table of Contents**

|  | | Page |
| --- | --- | --- |
| Supplementary Methods | Definitions of Pre- and Post-PCI Intravascular Imaging Measurements | 2-3 |
| Supplementary Table 1. | Peri-procedural complications | 5 |
| Supplementary Table 2. | Two-year clinical endpoints | 6 |
| Supplementary Table 3. | Baseline demographic and clinical characteristics after propensity score matching analysis | 7-9 |
| Supplementary Figure 1. | Kaplan Meier Curve After Propensity Score Matching for MACE | 10 |
| Supplementary Figure 2 | Kaplan Meier Curve After Propensity Score Matching for components of MACE and TVR | 11 |
| References |  | 12 |

**Definitions of Pre- and Post-PCI Intravascular Imaging Measurements**

**Pre-stent implantation assessments:(1)**

-Proximal/distal reference area: the site with the largest lumen area, proximal/distal to a stenosis but within the same segment (usually within 10 mm of the stenosis with no major intervening branches)

-Minimum luminal area (MLA): the smallest area bounded by the luminal border within the lesion site

-Average reference lumen area: (proximal + distal reference areas)/2

-Degree of stenosis (%): (Average reference lumen area – MLA)/ average reference lumen area

-Dominant lesion tissue characterization: lipid plaques appear as low-signal intensity regions with diffuse borders, fibrous plaques appear as high signal-intensity tissue, calcified plaques appear as low-signal intensity areas with sharply delineated borders, and thrombi appear as an intramural mobile mass attached to the luminal surface or floating within the lumen

**Post-stent implantation assessments:**

-Minimum Stent Area (MSA): the smallest area bounded by the stent border within the stented segment.(2)

-Stent Expansion Index (SEI) (%): the minimum stent area compared to the average reference lumen area after stenting.(3)

-Major dissection: the presence of residual plaque burden, extensive lateral (>60°) and longitudinal extension (>2 mm), involvement of deeper layers (medial or adventitia), and localization distal to the stent.(4)

-Stent malapposition: the axial distance between stent struts and vessel wall ≥0.4 mm with longitudinal extension ≥1 mm.(4)

-Stent struts protrusion: as a metallic neocarina where stent struts proximally extend the native bifurcation carina.(5)

**Supplementary Table 1. Peri-procedural complications**

|  | **JOS (112)** | **COS (60)** | ***P* value** |
| --- | --- | --- | --- |
| Peri-procedural MI**,** N (%)  Coronary no-reflow (TIMI<III)**,** N (%)  Distal wire perforation**,** N (%)  Vessel perforation**,** N (%)  Side branch occlusion**,** N (%) | 0 (0)  7 (6)  1 (1)  0 (0)  0 (0) | 0 (0)  6 (10)  0 (0)  0 (0)  0 (0) | NA  0.38  0.46  NA  NA |

Values are N (%) or Median (Interquartile range). *MI* myocardial infarction; TIMI thrombolysis in myocardial infarction

**Supplementary Table 2. Two-year clinical endpoints**

|  | **JOS (112)** | **COS (60)** | ***P* value** |
| --- | --- | --- | --- |
| **MACE** | 19 (17) | 14 (23) | 0.31 |
| Any death | 13 (12) | 8 (13) | 0.74 |
| Any MI | 0 (0) | 2 (3) | 0.052 |
| Any revascularization | 6 (5) | 7 (12) | 0.14 |
| Cardiovascular death | 11 (10) | 6 (10) | 0.97 |
| Non-cardiovascular death | 2 (2) | 2 (3) | 0.52 |
| TVMI | 0 (0) | 2 (3) | 0.052 |
| Non-TVMI | 0 (0) | 0 (0) | NA |
| TLR | 3 (3) | 4 (7) | 0.21 |
| TVR | 6 (5) | 7 (12) | 0.14 |
| Stent thrombosis | 0 (0) | 1 (2) | 0.17 |
| Any stroke | 2 (2) | 4 (7) | 0.10 |

Values are N (%), or Median (Interquartile range). *MACE* major adverse cardiac event; *TLR t*arget lesion revascularization; *TVMI t*arget vessel myocardial infarction; *TVR* target vessel revascularization.

**Supplementary Table 3. Baseline demographic and clinical characteristics after propensity score matching analysis**

|  |  | **Before PSM** |  |  |  |  | **After PSM** |  |  |
| --- | --- | --- | --- | --- | --- | --- | --- | --- | --- |
|  | **JOS (112)** | **COS (60)** | ***P* value** | **SMD** |  | **JOS (56)** | **COS (56)** | ***P* value** | **SMD** |
| Age (years) | 73 (66-80) | 77 (64-81) | 0.52 | 0.036 |  | 73 (66-80) | 77 (64-81) | 0.72 | 0.028 |
| Male sex | 78 (70) | 50 (83) | 0.050 | 0.314 |  | 46 (82) | 46 (82) | 1.00 | 0.000 |
| BMI | 22.6 (20.7-25.4) | 23.8 (21.7-25.7) | 0.11 | 0.144 |  | 23.2 (21.8-25.7) | 23.9 (21.5-25.9) | 0.81 | 0.033 |
| Hypertension | 78 (70) | 46 (77) | 0.33 | 0.157 |  | 38 (68) | 43 (77) | 0.29 | 0.200 |
| Diabetes mellitus | 51 (46) | 31 (52) | 0.44 | 0.123 |  | 26 (46) | 29 (52) | 0.57 | 0.107 |
| Dyslipidemia | 68 (61) | 40 (67) | 0.44 | 0.123 |  | 37 (66) | 37 (66) | 1.00 | 0.000 |
| Smoking  Never  Current  Former | 58 (52)  25 (22)  29 (26) | 30 (50)  8 (13)  22 (37) | 0.20 | 0.036  0.236  0.234 |  | 24 (43)  12 (21)  20 (36) | 28 (50)  8 (14)  20 (36) | 0.58 | 0.144  0.187  0.000 |
| Family history | 25 (22) | 19 (32) | 0.18 | 0.214 |  | 11 (20) | 17 (30) | 0.19 | 0.247 |
| Peripheral arterial disease | 3 (3) | 1 (2) | 0.68 | 0.067 |  | 2 (4) | 1 (2) | 0.56 | 0.111 |
| Hemodialysis | 8 (7) | 3 (5) | 0.58 | 0.088 |  | 2 (4) | 3 (5) | 0.65 | 0.086 |
| Prior MI | 10 (9) | 11 (18) | 0.73 | 0.287 |  | 9 (16) | 9 (16) | 1.00 | 0.000 |
| Prior PCI | 19 (17) | 18 (30) | 0.047 | 0.040 |  | 16 (29) | 15 (27) | 0.83 | 0.040 |
| Prior heart failure | 4 (4) | 2 (3) | 0.94 | 0.270 |  | 0 (0) | 2 (4) | 0.15 | 0.270 |
| Clinical presentation  CCS  UA  NSTEMI  STEMI | 40 (36)  8 (7)  12 (11)  52 (46) | 29 (48)  4 (7)  10 (17)  17 (28) | 0.11  0.91  0.27  0.021 | 0.258  0.019  0.174  0.381 |  | 25 (45)  3 (5)  9 (16)  20 (36) | 26 (46)  4 (7)  10 (18)  16 (29) | 0.85  0.70  0.80  0.42 | 0.036  0.074  0.048  0.153 |
| Cardiogenic shock | 9 (8) | 4 (7) | 0.77 | 0.052 |  | 4 (7) | 4 (7) | 1.00 | 0.000 |
| LV ejection fraction (%) | 49.2 (41.5-57.0) | 56.7 (44.3-58.7) | 0.027 | 0.314 |  | 53.5 (46.6-57.2) | 56.6 (41.6-58.7) | 0.49 | 0.038 |
| **Lab. Investigations**  Hemoglobin (g/dL)  Platelets (10^4/µL)  Creatinine (mg/dL)  HbA1c (%)  LDL-c (mg/dL) | 13.4 (12.0-15.0)  20.0 (16.1-24.6)  0.90 (0.78-1.20)  6.0 (5.6-6.9)  98 (69-125) | 13.6 (12.1-14.6)  20.0 (16.3-25.6)  0.96 (0.80-1.20)  6.2 (5.7-7.0)  91 (67-122) | 0.87  0.92  0.33  0.32  0.23 | 0.000  0.016  0.094  0.087  0.216 |  | 13.8 (12.1-15.0)  19.7 (15.5-24.4)  0.96 (0.81-1.25)  6.2 (5.6-7.2)  92.5 (66.3-119.0) | 13.6 (12.1-14.6)  20.3 (15.9-25.7)  0.96 (0.82-1.23)  6.2 (5.7-7.0)  91.0 (67.8-122.8) | 0.53  0.41  0.89  0.83  0.95 | 0.104  0.180  0.252  0.000  0.013 |
| **Discharge medications**  Aspirin  P2Y12 inhibitors  ACEIs/ARBs  BBs  Lipid lowering drugs  Nitrates  SGLT2i  CCBs  OAC | 110 (98)  111 (99)  86 (77)  75 (67)  111 (99)  13 (12)  12 (11)  28 (25)  8 (7) | 58 (97)  60 (100)  44 (73)  31 (52)  59 (98)  9 (15)  11 (18)  12 (20)  4 (7) | 0.30  0.46  0.62  0.41  0.65  0.53  0.16  0.46  0.91 | 0.103  0.117  0.080  0.315  0.072  0.102  0.224  0.118  0.019 |  | 56 (100)  56 (100)  43 (77)  33 (59)  55 (98)  10 (18)  8 (14)  13 (23)  2 (4) | 55 (98)  56 (100)  40 (71)  29 (52)  55 (98)  9 (16)  9 (16)  11 (20)  3(5) | 0.32  1.00  0.52  0.45  1.00  0.80  0.79  0.65  0.65 | 0.190  0.000  0.122  0.144  0.000  0.048  0.050  0.087  0.086 |

Values are N (%) or Median (Interquartile range). *ACEIs* angiotensin converting enzyme Inhibitors; *ARBs* angiotensin II receptor blockers; *BBs* beta blockers; *BMI b*ody mass index; *CCBs* calcium channel blockers; *CCS* chronic coronary syndrome; *HbA1c* glycated hemoglobin; *LDL-C* low-density lipoprotein cholesterol; *MI* myocardial infarction; *NSTEMI* Non-ST elevation myocardial infarction; O*AC* oral anticoagulation; *PCI p*ercutaneous coronary intervention; *SGLT2i* sodium glucose co-transport 2 inhibitors; *STEMI* ST elevation myocardial infarction; *UA* unstable angina.

**Supplementary Figure 1**

**Title:** Kaplan-Meier Curve After Propensity Score Matching for MACE

**Caption:** *CI* confidence interval; *COS* crossover stenting; *HR* hazards ratio; *MACE* major adverse cardiac event; *JOS* just ostial stenting.

**Supplementary Figure 2
Title:** Kaplan-Meier Curve After Propensity Score Matching for Components of MACE and TVR

**Caption:** (A) Any Death, (B) Any MI, (C) Any revascularization, (D) TVR. *CI* confidence interval; *COS* crossover stenting; *HR* hazards ratio; *MACE* major adverse cardiac event; *MI* Myocardial infarction; *TVR* target vessel revascularization; *JOS* just ostial stenting.

**References**:

1.	Fujii K, Kubo T, Otake H, Nakazawa G, Sonoda S, Hibi K, Shinke T, Kobayashi Y, Ikari Y, Kozuma K, Akasaka T. Expert consensus statement for quantitative measurement and morphologic assessment of optical coherence tomography: update 2025. Cardiovasc Interv Ther. 2025;40(2):226-33.

2.	Mintz GS, Lefèvre T, Lassen JF, Testa L, Pan M, Singh J, Stankovic G, Banning AP. Intravascular ultrasound in the evaluation and treatment of left main coronary artery disease: a consensus statement from the European Bifurcation Club. EuroIntervention. 2018;14(4):e467-e74.

3.	Rai H, Harzer F, Otsuka T, Abdelwahed YS, Antuna P, Blachutzik F, Koppara T, Raber L, Leistner DM, Alfonso F, Nef H, Seguchi M, Aytekin A, Xhepa E, Kufner S, Cassese S, Laugwitz KL, Byrne RA, Kastrati A, Joner M. Stent Optimization Using Optical Coherence Tomography and Its Prognostic Implications After Percutaneous Coronary Intervention. J Am Heart Assoc. 2022;11(9):e023493.

4.	Raber L, Mintz GS, Koskinas KC, Johnson TW, Holm NR, Onuma Y, Radu MD, Joner M, Yu B, Jia H, Meneveau N, de la Torre Hernandez JM, Escaned J, Hill J, Prati F, Colombo A, Di Mario C, Regar E, Capodanno D, Wijns W, Byrne RA, Guagliumi G. Clinical use of intracoronary imaging. Part 1: guidance and optimization of coronary interventions. An expert consensus document of the European Association of Percutaneous Cardiovascular Interventions. EuroIntervention. 2018;14(6):656-77.

5.	Lunardi M, Louvard Y, Lefevre T, Stankovic G, Burzotta F, Kassab GS, Lassen JF, Darremont O, Garg S, Koo BK, Holm NR, Johnson TW, Pan M, Chatzizisis YS, Banning A, Chieffo A, Dudek D, Hildick-Smith D, Garot J, Henry TD, Dangas G, Stone GW, Krucoff MW, Cutlip D, Mehran R, Wijns W, Sharif F, Serruys PW, Onuma Y, Bifurcation Academic Research C, European Bifurcation C. Definitions and Standardized Endpoints for Treatment of Coronary Bifurcations. J Am Coll Cardiol. 2022;80(1):63-88.
